# Comparing DNA, RNA and protein levels for measuring microbial dynamics in soil microcosms amended with nitrogen fertilizer

**DOI:** 10.1038/s41598-019-53679-0

**Published:** 2019-11-26

**Authors:** Luis H. Orellana, Janet K. Hatt, Ramsunder Iyer, Karuna Chourey, Robert L. Hettich, Jim C. Spain, Wendy H. Yang, Joanne C. Chee-Sanford, Robert A. Sanford, Frank E. Löffler, Konstantinos T. Konstantinidis

**Affiliations:** 1 School of Civil and Environmental Engineering, Georgia Institute of Technology, Atlanta, Georgia USA; 2 Chemical Sciences Division, Oak Ridge National Laboratory, Oak Ridge, Tennessee USA; 3 Graduate School of Genome Science and Technology, University of Tennessee, Knoxville, Tennessee USA; 4 Center for Environmental Diagnostics & Bioremediation, University of West Florida, Pensacola, Florida USA; 5 Department of Geology, University of Illinois at Urbana-Champaign, Urbana, Illinois USA; 6 Department of Plant Biology, University of Illinois at Urbana-Champaign, Urbana, Illinois USA; 7 U.S. Department of Agriculture, Agricultural Research Service, Urbana, Illinois USA; 8 Center for Environmental Biotechnology, Department of Microbiology, Department of Civil and Environmental Engineering, and Department of Biosystems Engineering and Soil Science, University of Tennessee, Knoxville, Tennessee USA; 9 Biosciences Division, Oak Ridge National Laboratory, Oak Ridge, Tennessee USA; 10 Present Address: Laboratorio de Enteropatogenos, Programa de Microbiología y Micología, ICBM, Facultad de Medicina, Universidad de Chile, Santiago, Chile

**Keywords:** Element cycles, Soil microbiology, Metagenomics

## Abstract

To what extent multi-omic techniques could reflect *in situ* microbial process rates remains unclear, especially for highly diverse habitats like soils. Here, we performed microcosm incubations using sandy soil from an agricultural site in Midwest USA. Microcosms amended with isotopically labeled ammonium and urea to simulate a fertilization event showed nitrification (up to 4.1 ± 0.87 µg N-NO_3_^−^ g^−1^ dry soil d^−1^) and accumulation of N_2_O after 192 hours of incubation. Nitrification activity (NH_4_^+^ → NH_2_OH → NO → NO_2_^-^ → NO_3_^−^) was accompanied by a 6-fold increase in relative expression of the 16S rRNA gene (RNA/DNA) between 10 and 192 hours of incubation for ammonia-oxidizing bacteria *Nitrosomonas* and *Nitrosospira*, unlike archaea and comammox bacteria, which showed stable gene expression. A strong relationship between nitrification activity and betaproteobacterial ammonia monooxygenase and nitrite oxidoreductase transcript abundances revealed that mRNA quantitatively reflected measured activity and was generally more sensitive than DNA under these conditions. Although peptides related to housekeeping proteins from nitrite-oxidizing microorganisms were detected, their abundance was not significantly correlated with activity, revealing that meta-proteomics provided only a qualitative assessment of activity. Altogether, these findings underscore the strengths and limitations of multi-omic approaches for assessing diverse microbial communities in soils and provide new insights into nitrification.

## Introduction

Even though the central role of microbes in the cycling of nitrogen is recognized, the dynamics and controls of the interrelated microbial nitrogen pathways in agricultural soils are not completely understood. This knowledge gap limits the development of accurate, predictive models of nitrogen flux that encompass the role of microbes in the generation and consumption of nitrogen substrates, as well as the emission of greenhouse gases, including nitrous oxide (N_2_O)^1^. In agricultural soils receiving large inputs of nitrogen fertilizer, nitrifiers such as ammonia-oxidizing bacteria (AOB), ammonia-oxidizing archaea (AOA) and nitrite-oxidizing bacteria (NOB) collectively are responsible for the conversion of ammonium to nitrate^2,3^. In addition, the recent discovery of *Nitrospira* bacteria capable of complete oxidation of ammonia to nitrate (comammox) has suggested that the process of nitrification in natural environments might be carried out by a single taxon^4,5^. Under anoxic conditions (e.g., water saturated soils), nitrate (NO_3_^−^) can be reduced to gaseous forms such as dinitrogen (N_2_), nitric oxide (NO) or N_2_O by denitrifying organisms and consequently be lost to the atmosphere. It has also been reported that nitrification is a major N_2_O source under low oxygen concentrations^3,6^, although detailed mechanistic understanding is lacking^7^. Despite the importance of nitrification in the generation of N_2_O and NO_3_^−^, the relative contributions of AOA, AOB and NOB populations in this process, especially during soil fertilization events, are still subjects of intensive research^8^, and the relative contribution of the comammox bacteria to the process is not clear^9^. Thus, understanding the niche specialization and diversity of nitrifiers in terrestrial ecosystems is essential for better prediction of the contributions of these microbial taxa to the nitrogen cycle and the modeling of the corresponding activities and products. High-throughput sequencing and proteomic approaches can characterize the diversity of nitrogen pathways in the environment. However, to what extent these omic approaches could also reflect microbial activity is less clear.

Although DNA, RNA, and protein abundances all reflect microbial potential and responses to environmental changes and thus, could be used to study nitrogen cycling in soils, each measurement generally offers different types of information. For instance, metagenomics (DNA level) offers a comprehensive overview of the functional potential of microbial communities but does not reflect active community members or functions. Short-term microbial responses to external changes (e.g., nitrogen addition) can be tracked by analyzing the actively expressed genes (i.e., metatranscriptomics). For instance, the relationship between measured nitrification processes and the ammonia monooxygenase (*amoA*) transcripts have revealed differences between archaeal and bacterial activity in acidic soils^10^. Proteomics provides a third level of molecular information by reflecting synthesized enzymes that catalyze reactions. Although proteomics has been applied to only a limited number of natural microbial communities, the results have provided new insights about metabolic pathways and interdependencies among microbial groups [reviewed before^11^]. However, most of these advances are hindered by the intrinsic complexity of soils. For instance, soil samples are challenging to analyze not only because of their heterogeneous structure and chemical composition (e.g., low quality and quantity of extracted nucleic acids), but also because of the highly diverse microbial communities and slow growth kinetics. Nonetheless, recent advances in metagenomic and metaproteomic techniques as well as integration with stable isotope probing (SIP) have helped elucidate the role of previously elusive keystone microbial populations^12^. For instance, the combination of multi-omic datasets provided new insights into diversity and gene potential of microbial communities of permafrost ecosystems, but the datasets were less predictive of measured process rates^13^.

Toward closing the abovementioned knowledge gaps, we examined nitrogen-amended sandy soils obtained from a site with a history of agricultural management and application of synthetic nitrogen fertilizer. A prior year-round characterization of field samples from the same agricultural site revealed increased abundance of novel *Thaumarchaeota* and comammox nitrifiers, but the findings were limited to metagenomics^14^. Here, our goal was to assess the strengths and limitations of multi-omics in detecting microbial activity by correlating measurements of DNA, RNA, and protein abundances with measured rates of nitrate formation and N_2_O production in soils incubated under controlled conditions in the laboratory. The results revealed that metatranscriptomic data best reflected the measured nitrification rates under the tested experimental conditions and provided novel insights about nitrifier gene expression dynamics after a simulated nitrogen fertilization event.

## Results

### Nitrification activity in soil microcosms

**Figure 1:**
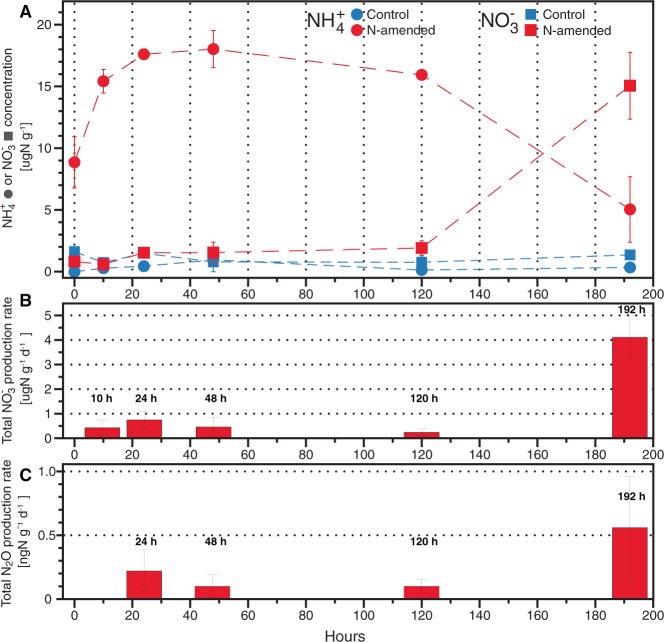
Nitrogen pools and fluxes in soil incubations amended with NH_4_^+^ and urea. Mean NH_4_^+^ and NO_3_^−^ concentrations (**A**), total NO_3_^−^ production rate (**B**), and total N_2_O production rate (**C**) for the nitrogen-amended microcosms at each incubation time point. Error bars represent the standard deviation from replicate samples (n = 6 for nitrogen-amended and n = 3 for control).

Patterns in nitrification rates were consistent with NO_3_^−^ formation and NH_4_^+^ disappearance during an eight-day period following the amendment of soil microcosms with an equimolar nitrogen mixture of NH_4_^+^ and urea, representative of fertilizer application in the field. Nitrate was not supplied as part of the amendment because it could represent both product and substrate for different nitrogen cycle pathways. Based on the NH_4_^+^ concentration patterns, urea quickly hydrolyzed to release NH_4_^+^ within the first two days of incubation (Fig. 1a). Specifically, the NH_4_^+^ concentrations peaked at 48 hours of incubation (avg = 18.02 and SD =  ± 1.5 µgN-NH_4_^+^ g^−1^ dry soil) from urea hydrolysis, and decreased to 5.4 ± 2.5 µgN-NH_4_^+^ g^−1^ dry soil by 192 hours of incubation. Consistent with the disappearance of NH_4_^+^, the NO_3_^−^ concentrations increased from an initial value of 0.81 ± 0.28 µgN-NO_3_^-^ g^−1^ dry soil to 1.91 ± 0.5 µgN-NO_3_^-^ g^−1^ dry soil at 120 hours of incubation, and then increased at a faster rate to 15.06 ± 2.7 µgN-NO_3_^−^ g^−1^ dry soil at 192 hours of incubation (Fig. 1a). Measured nitrate accumulation and nitrification rates confirm that nitrification activity was relatively low (<1 µgN-NO_3_ g^−1^ dry soil) for the first 120 hours of incubation and increased five to eight days after the addition of NH_4_^+^ and urea mixtures, reaching an average nitrification rate of 4.1 ± 0.87 µg N-NO_3_^−^ g^−1^ dry soil d^−1^ (n = 6) after 192 hours of the incubation (Fig. 1b). As a result of nitrification activity, pH values decreased across replicated nitrogen-amended microcosms during the incubation (Table S1). In order to examine the generation of N_2_O possibly generated as a by-product of oxidation reactions during the incubation, we measured the production of N_2_O in nitrogen-amended incubations. Net N_2_O production rates in the incubation headspace increased from 0.22 ± 0.16 ng N-N_2_O g^−1^ dry soil d^−1^ after 24 hours to 0.56 ± 0.40 ng N-N_2_O g^−1^ dry soil d^−1^ at the end of the incubations (Fig. 1c). Control microcosms receiving only irrigation water (i.e., no nitrogen amendment) did not show net NH_4_^+^oxidation.

To evaluate possible differences between the use of NH_4_^+^ or urea in nitrifying activity, we examined patterns in ^15^N-NO_3_^−^ production rates in microcosms that received ^15^N-labeled NH_4_^+^ versus ^15^N-labeled urea by tracing the fate of 15 N label in the fertilized treatments. In general, ^15^NO_3_^−^ production was similar between ^15^NH_4_^+^ and ^15^N-urea microcosms, although rates were higher after 10 hours in ^15^NH_4_^+^ microcosms and 48 hours in ^15^N-urea microcosms (two tailed *t*-test, *P* < 0.01), but converged thereafter (Fig. S1). By the end of the incubations, approximately half of the added ^15^N was converted to ^15^N-NO_3_^−^ (49–55% for both labelled solutions/treatments), only a small fraction was converted to ^15^N-N_2_O (0.006–0.01%), and a large percentage remained as ^15^N-NH_4_^+^ (19 ± 11%) (Fig. S1). The remaining added nitrogen was presumably rapidly lost as N gas, assimilated into microbial biomass, or adsorbed to soil particles, all well-known nitrogen sinks of ^15^N tracer studies in soils^15,16^.

### Taxonomy of microbial soil populations based on 16S rRNA gene sequences

The taxonomic composition and abundances of the main microbial groups determined from recovered 16S rRNA gene sequences (DNA level) from nitrogen-amended incubations, were generally stable during incubations (Fig. S2). In agreement with our previous results based on field samples from the same agricultural site^14^, bacterial and archaeal groups associated with nitrification were comparatively less abundant than (non-nitrifer) abundant bacterial taxa (e.g., *Actinobacteria*, *Flavobacteria*, and *Acidobacteria*) in both DNA and cDNA datasets. For instance, known AOB and NOB genera such as *Nitrosomonas* and *Nitrospira* had average relative abundances of 0.01% and 1.6% of the total populations in the metagenomes from incubated soils. Additionally, the relative abundances of the AOA genera related to *Nitrososphaera* and *Nitrosopumilus* were 0.9% and 0.3% in the microcosm metagenomes. We note however that due to the high coverage obtained by our datasets, the 16S rRNA gene (and protein-coding genes; see below) of these nitrifiers were adequately sampled. Notably, the 16S rRNA gene transcript abundances for AOB conspicuously increased during the incubation period (Fig. S3). In fact, relative 16 S rRNA gene expression ratios (cDNA/DNA) for AOB and NOB belonging to *Nitrosospira*, *Nitrosomonas* and *Nitrospira* increased 3-,6-, and 14-fold between 10 and 192 hours. In contrast, the 16S rRNA gene expression ratios for the archaeal groups *Nitrososphaera* and *Nitrosopumilus* were stable during the same incubation period, although with ~50% increase in relative expression at 48 h of incubation (Fig. S3). A description of detected functions in metatranscriptomes is available in the Supplementary Material. We sought to examine next the dynamics of individual populations/genomes.

### Individual populations from microcosm metagenomes

Given that none of the recovered metagenome-assembled genomes (MAGs) represented AOA, AOB, NOB, or comammox populations, we included MAGs obtained from a previous analysis of field samples from the same site (Havana county, Illinois, USA) and depth as the soil used in the soil microcosms in the present study^14^. MAGs potentially involved in nitrification processes were likely missed in the microcosm metagenomes due to comparatively lower sequencing effort than the field samples and the relatively low abundances of these groups. The MAGs (designated with the letter F at the end of their name for Field metagenomes) consisted of two complete ammonia oxidizer (comammox) *Nitrospira* MAGs (MAG021F and MAG017F) and five ammonia-oxidizing archaea MAGs representing the *Thaumarchaeota* lineages I.1b (MAG032F and MAG019F) and I.1a (MAG004F, MAG109F, and MAG001F) (Fig. S4b). The genetic relatedness of the field MAGs compared to the populations found in the microcosm metagenomes based on the identity of reads^17^ from latter metagenomes recruited against these MAGs was high (ANIr avg = 99.3, SD = 0.27). These results confirmed that the MAGs from the field metagenomes well represented the populations in the microcosms (Fig. S4c). These seven nitrifier MAGs recruited between 0.36% to 0.77% of the microcosm metagenomic reads in each dataset, with an average of 0.55%, and between 0.46% and 0.92% of the metatranscriptomic libraries, which was comparable to the 16S rRNA gene-based abundances mentioned above. This level of relative abundance was adequate for assessing the gene and transcript dynamics of the MAGs since it provided, on average, more than 7.5X coverage of the corresponding gene sequences (i.e., the time each base is covered by transcriptomic reads). Additional statistics for metagenomes and metatranscriptomes datasets, including sequencing effort, coverage obtained, and statistics of all recovered MAGs are available in the Supplementary Material and Tables S2–5.

Relative expression values of MAGs (measured as transcriptomic reads per kilobase million, RPKM) were used as a proxy for comparing the response and metabolic activity among nitrifying bacteria and archaea between samples (incubation time points). Even though expression values for most nitrifying MAGs belonging to *Nitrospira* and *Thaumarchaeota* were stable and relatively low, AOA MAG004F, MAG019F and comammox MAG017F had, on average, the highest expression values throughout the incubations (Fig. S4a). For instance, the increase in expression values for AOA belonging to the I.1b clade, MAG004F and MAG032F, were 39% and 50% after 48 hours of incubation (compared to expression levels at 10 hours incubation), respectively. In contrast, gene expression of comammox MAG017F increased by 59% after 120 and 192 hours of incubation (Fig. S4a). Note that AOB and NOB were not included in the RPKM analysis due to lack of recovered MAGs representing these populations (see above). Given the technical limitations in recovering high quality nitrifier MAGs, a gene-based approach was also employed in order to assess changes in gene fragments (DNA) and transcript (cDNA) abundances of genes involved in nitrification activity for AOB and NOB nitrifiers (see below).

### Quantification of nitrification genes in microcosms

**Figure 2:**
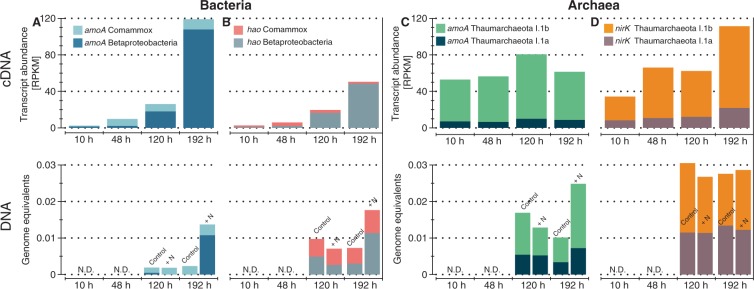
Nitrification genes in incubated soils. Transcript abundance (RPKM, top panel) and gene abundance (genome equivalent, lower panel) for bacterial *amoA* (**A**), *hao* (**B**), thaumarchaeotal *amoA* (**C**), and *nirK* (**D**) are shown. Transcript or DNA abundances for specific clades are showed in different colors.

To further explore the microbial nitrification processes in incubated soils at the gene level, we specifically quantified gene fragments and transcripts directly involved in nitrification reactions using ROCker, a tool developed to accurately differentiate between reads encoding different gene families or (distinct) phylogenetic clades within a gene family^18^ (see Methods for details). Relative expression values belonging to the gene encoding urease subunit c (*ureC*) were relatively high after 48 and 192 h of incubation but the average abundances were lower compared to other nitrification genes (e.g., *amoA*; see Fig. S5a). For instance, the relative expression of the bacterial gene encoding ammonia monooxygenase subunit alpha (*amoA*) was 53.5-fold higher compared to the expression values at 10 h of incubation. Most of the detected *amoA* transcripts (cDNA) were phylogenetically affiliated with *Betaproteobacteria* and corresponded to up to 90% of the total detected bacterial *amoA* transcripts at 192 hours of incubation (Fig. 2a). Notably, regression analysis between *amoA* transcript abundances and measured nitrate concentrations at 10, 48, 120 and 192 h of incubation had a strong relationship (Coefficient of determination, *r*^2^ > 0.97, Fig. S5c). The *r*^2^ values determined for transcripts belonging to other nitrification genes during the same incubation points were lower and ranged from ~0 to 0.7. Similarly, low *r*^2^ values were observed when using the DNA level (i.e., metagenomic reads) for other nitrification genes. After 192 hours of incubation, betaproteobacterial *amoA* transcript abundance increased 66-fold, whereas comammox *amoA* gene transcripts remained stable (Fig. 2a). The latter results indicated that the comammox *amoA* may be more abundant under field conditions but betaproteobacterial *amoA* might show a faster response upon ammonia addition, which was also consistent with a previous study^14^.

Although the relative expression for the archaeal *amoA* was more stable throughout the incubation compared to its betaproteobacterial counterparts, maximum expression was reached after 120 hours of incubation, suggesting that archaeal AmoA activity temporarily increased at later time points during the incubation. Archaeal *amoA* transcripts belonging to the group I.1b were ~7 times more abundant than their I.1a counterpart across the incubations (Fig. 2c). Similar to *amoA* patterns, the relative expression for the betaproteobacterial hydroxylamine oxidoreductase (*haoA;* NH_2_OH → NO_2_^−^) steadily increased during the incubations, whereas comammox *haoA* transcripts were stable throughout the incubations (Fig. 2b). Expression values for the nitrite oxidoreductase subunit alpha (*nxrA;* NO_2_^−^ → NO_3_^−^) had a 12.4-fold increase compared to the 10-hour time point, consistent with the patterns observed for the previous nitrification genes and NO_3_^−^ accumulation (Fig. S5a). Unexpectedly, expression values for *nirK* (NO_2_^−^ → NO) affiliated to *Thaumarchaeota* were higher compared to *nirK* transcripts assigned to the *Nitrospira* clade (Fig. 2d). In fact, *Thaumarchaeota nirK* transcripts had a 3.4-fold increase after 192 hours of incubation relative to earlier sampling points, indicating that *Thaumarchaeota* might have been more active in the reduction of nitrite compared to other steps of nitrification. Specifically, there was a 3.4-fold increase for clade I.1b *nirK* transcripts during the 10 to 192 hours of incubation period, whereas the abundance of transcripts from clade I.1a were stable throughout the incubations (Fig. 2d). We were also able to assign several of the gene sequences used above to individual MAGs using a phylogenetic approach. Consistent results with those reported above were observed when examining MAG-specific expression patterns. For instance, a high fraction of the total comammox *hao* and thaumarchaeotal *nirk* transcripts, ranging from ~62 to 82%, were assigned to MAGs. However, the majority of detected *amoA* and *hao* transcripts were derived from soil betaproteobacterial AOB communities (Fig. 2a) not represented by the recovered MAGs (i.e., these genes were part of the un-binned soil predicted sequences). Thus, a MAG-centered approach would have missed the activity of these communities since no betaproteobacterial AOB MAGs were recovered.

In summary, the metatranscriptomic profiles suggested that AOB, but not comammox, responded rapidly to the nitrogen amendment, whereas AOA transcriptome shifts were less pronounced. The response of AOB, and to a lesser extent AOA, was also reflected at the DNA level, albeit with a substantial time delay (Fig. 2, lower panels). For instance, shifts were observed early at the transcript level while at the DNA level changes were mostly evident 192 hours after the start of incubation (Fig. 2a,b). One exception was the urease gene (*ureC*) that showed high abundance at all incubation time points although its transcription response was lower compared to nitrification genes (Fig. S5a,b). Nonetheless, these results were consistent across the individual nitrification steps and indicated that at least the AOB nitrifiers grew in response to nitrogen addition.

### A proteomic perspective in soil microcosms

**Figure 3:**
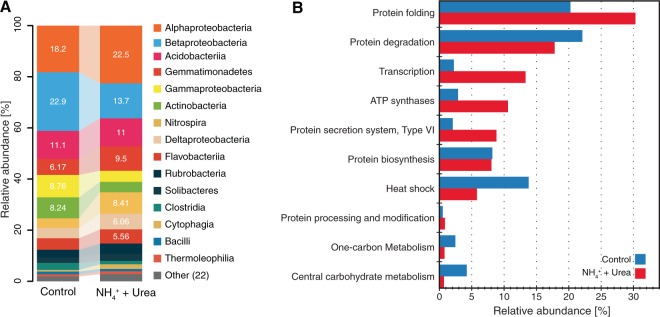
Metaproteomic analyses of incubated soils at 192 hours of incubation. Panel (A) shows taxonomic affiliation (class) and abundance (average spectral counts) for peptides detected in control and N-amended incubations. Panel (B) shows summarized functional annotation of detected peptides using SEED functional categories.

A metaproteomic analysis of the control and nitrogen-amended microcosms at 192 hours of incubation detected a total of 2,892 and 1,629 non-redundant peptides, respectively. A total of 844 peptides were shared among control and nitrogen-amended incubations, whereas 2,048 and 785 were exclusively present in each microcosm, respectively. Most of peptides detected in control and nitrogen-amended incubations matched protein sequences predicted from metagenomic assemblies (89.4% and 88.2%, respectively) and the remaining fraction matched reference proteomes (Table S6). The top 20 most abundant proteins in control and nitrogen-amended treatment microcosms were related to housekeeping and transport proteins whereas in the latter incubation, oxidoreductases for small carbon and alcohol molecules and ATP synthesis were among the most abundant proteins detected (Table S7). These results were also consistent for some of the highly expressed genes in metatranscriptomes related to protein and RNA metabolism at 192 h of incubation (See Supplementary Material). The taxonomic affiliation, at the class level, for the most relatively abundant annotated peptides belonged to *Alphaproteobacteria*, *Betaprotebacteria*, and *Acidobacteria* in control and nitrogen-amended incubations. Although there were major differences in abundances for groups such as *Betaproteobacteria* (40% decrease) and *Gammaproteobacteria* (50% decrease) (Fig. 3a), higher abundances were detected for less abundant groups commonly associated with the nitrification process. For instance, close to a 2.2-fold increased abundance for nitrogen-amended incubations were detected for peptides belonging to *Nitrospira*. Detected peptides related to folding and synthesis were the most abundant and had similar abundances in the control and nitrogen-amended microcosms after 192 hours of incubation. However, the relative abundance was higher for annotated functions related to ATP synthases and transcription categories in the nitrogen-amended samples relative to the control, presumably as a consequence of a higher microbial activity generated after the nitrogen input. On the other hand, heat-shock and degradation proteins were more abundant in the control incubation, probably reflecting a more prevailing dormant state for the microbial communities in these samples (Fig. 3b). However, unlike the metagenomic and metatranscriptomic datasets, only some peptides involved in nitrification were identified using metaproteomics. For instance, the detected peptides directly involved in nitrification pathways corresponded to the nitrite oxidoreductase subunit B (NxrB), which had a 31.3% abundance increase in the nitrogen-amended samples compared to the control.

## Discussion

### Using multi-omic approaches for examining process rates

Measuring nitrification rates in incubated soils allowed us to evaluate the explanatory and predictive power of omic approaches in a highly diverse soil system. Although all three omic approaches revealed increased abundance for target genes, transcripts, and proteins related to nitrification pathways, they differed in temporal resolution and detection capabilities. For instance, the strongest agreement to the observed nitrification processes (i.e., ammonia or nitrite oxidation) was for the metatranscriptomic data within the first days of incubations (e.g., Fig. 2a,b,d), whereas metagenomes lagged behind and only reflected the ongoing nitrification process after 192 hours of incubation (e.g., lower panels Fig. 2a,b). These data were presumably attributed to the fact that growth (e.g., at least a few replication cycles) should occur before metagenomics can reveal shifts in relative abundance over time. Note that microbial growth was not explicitly measured by our study to further corroborate the above interpretation. Therefore, metagenomics could also reflect ongoing microbial processes if the processes are ongoing for a period of time and are coupled with the growth of the corresponding organisms, even in soils. In contrast, if the goal is to see immediate responses to a perturbation or if the perturbation is short-lived (e.g., lasting a few hours), metatranscriptomic data will be preferable. We also observed that metatranscriptomes were as good as metagenomics, if not better, at reflecting microbial activity for nitrification processes even at later incubation time points. In contrast, the metaproteomes offered, at most, a qualitative glimpse at nitrification processes and were less definitive in identifying common nitrification markers. The latter was largely attributable to the computational challenges associated with proteomic data such as high peptide redundancy and the requirement of high-quality assemblies which are still challenging for highly complex soil metagenomes. Furthermore, many challenges remain for efficient extraction of membrane proteins from low abundance organisms such as nitrifiers. Ultimately, these technical limitations were reflected in a lower number of detected proteins compared to the number of metagenomic and metatranscriptomic reads recovered that encoded the proteins of interest in our datasets.

While shifts in 16S rRNA gene ratios (cDNA/DNA) were relatively small for AOA, the 16S rRNA and functional gene ratio shifts (e.g., *amoA*) for AOB/NOB were much more pronounced throughout the incubations (Fig. S3). These results might reflect an active and growing state for AOB/NOB and mostly active AOA communities as observed before for agricultural soil microcosms^19^. The differences observed between (high) target gene abundances and (low) 16S rRNA gene ratios for AOA could reflect a limitation of the latter approach when used as a proxy for assessing microbial activity (e.g., inconsistent correlation between rRNA and activity)^20^. Thus, targeting specific functional genes in AOA could offer an alternative approach for tracking their activity in metatranscriptomes. However, more frequent sampling and incubations under different physicochemical conditions will be required for more robust conclusions to emerge on the exact relationship(s) between rRNA marker abundances and process rates.

Future incubation studies could increase the number of samples analyzed and shed light on the intrinsic differences between nitrifier (and denitrifier) communities by testing variables such as oxygen availability (i.e., water saturation) and different agricultural soil types. For instance, the incubation conditions used in our study deliberately promoted nitrification over denitrification processes and as a result, the N_2_O production was detected due to the former process. Consequently, nitric oxide (e.g., *norB*) and nitrous oxide reductases (e.g., *nosZ*) transcripts, which are responsible for N_2_O production and consumption during denitrification, respectively, were not detected in our metatranscriptomes datasets (i.e., abundance below detection limit). Also, the use of nitrification inhibitors could help to elucidate the origin of the measured N_2_O whether production was biotic or abiotic, for which our data are limited in predicting.

Previous studies have also found metatranscriptomic approaches to be better predictors of measured microbial activity^21^ in controlled laboratory systems amended with exogenous organic compounds or natural communities, but have been more limited in providing insights into the whole-microbial community response to the amendment. For instance, a high correlation between environmental conditions and the expression of adaptation mechanisms (transcription level) was observed in acid mine drainage communities^22^. On the other hand, multi-omic approaches applied to permafrost microbial communities were less predictive of biogeochemical processes such as methane oxidation but provided a higher overview of active (transcription and proteomic levels) microbial members encoding these pathways^13^. Similar approaches led to the discovery of unexcepted microbial pathways such as the methanogenesis in oxygenated soils^23^. Here, our metatranscriptomic results were effective in reflecting measured nitrification even though our observations were based on single samples at different time points. Nonetheless, temporal data typically require less replication for statistically robust results and likely offset the experimental noise between sampling time points compared to cross-sectional data. Hence, our main conclusions were likely not affected by the relatively small number of samples analyzed. Taken all together, our analysis showed that metatranscriptomics could reliably and quantitively reflect undergoing microbial processes even in the soil microcosms which represent an intrinsically challenging matrix for RNA work compared to other ecosystems (e.g., aquatic environments^24^). It should be noted that different soil types may not be as amenable to perform RNA extractions (e.g., not enough high-quality RNA). Hence, metatranscriptomics may continue to be a challenging task for certain soil types and conditions.

### New insights into nitrification pathways

In terms of the ecological adaptation of the nitrifiers analyzed here, the Havana agricultural site has had a long history of cyclical seasonal inputs (e.g., fertilizers) that have shaped the structure of microbial communities differently between soil layers. The AOA and AOB communities in the Havana site have legacy establishments at the 20–30 cm soil depth and are under relatively stable environmental conditions compared to the top soil layer that receives most of the nitrogen fertilizer^14^. Thus, nitrogen amendments tested in our experiment and experimental conditions might not represent closely the conditions usually experienced by the examined, deep-layer AOA and AOB communities (20–30 cm). The rapid response of AOB observed here might be a reflection of physiological adaptations of AOB to thrive under high nitrogen content as reported previously^19^. In contrast, the low response observed for comammox and some AOA communities might reflect their limited physiological capabilities to respond to high nitrogen concentrations^4,5^ that were assayed in our experimental setup.

Assessing the individual gene level, as opposed to whole genome transcript level, provided more robust results for relating population response to measured nitrification reactions, presumably due to higher sequence coverage (less noise). Our results showed that even though betaproteobacterial *amoA* transcripts responded to the addition of ammonium and urea, the relative abundance of comammox *amoA* transcripts was stable (i.e., not responding to the nitrogen amendment), although comammox populations were relatively more abundant than AOB in the microcosms. This observation is consistent with previous metagenomic results from the same agricultural soil, where comammox *amoA* genes and the organisms encoding these genes represented the highest fraction of nitrifying bacteria^14^. The differences between measured genes and transcripts indicated that the incubation conditions favored the activity of *Betaproteobacteria* over comammox nitrifying bacteria, suggesting ecophysiological differences among these taxa for the incubation conditions or added substrates compared to field conditions.

The sequencing of isolates and environmental AOA genomes has shown that even though they encode an AmoA protein, they lack a canonical hydroxylamine oxidation pathway^25^. Previous studies have proposed that nitric oxide is essential for hydroxylamine oxidation to nitrite in archaea^26^. The proposed mechanism involves oxidation of ammonium to hydroxylamine followed by oxidation to nitrite catalyzed by a putative Cu-protein that uses nitric oxide as co-reactant for the oxidation of hydroxylamine. Interestingly, nitric oxide has been proposed to be derived from the activity of the NirK enzyme present in most AOA sequenced genomes. Our results show that unlike AOA *amoA* or bacterial *nirK* transcripts, *Thaumarchaeota nirK* transcripts increased in abundance in the incubated soils, supporting the abovementioned hypothesis. Therefore, even though AOA *amoA* transcripts did not show clear changes in abundances compared to their bacterial counterparts, these results agree with the previous observations in marine and terrestrial systems^26,27^, and likely denote an unaccounted role for *Thaumarchaeota nirK* in nitrification in agricultural soils.

### Challenges and opportunities for multi-omic studies

Here we analyzed total RNA extractions from soils where ribosomal RNA transcripts represented 94–98% of the total sample, limiting our study to a small fraction of transcripts related to functional genes. Current experimental approaches offer successful rRNA depletion for environmental samples, when RNA yields are not limiting^28^, which was not the case in our study. Additionally, all the results represented here provided only relative abundances for measured microbial markers. For instance, approaches such as qPCR or internal standards spiked into the DNA or cDNA library for sequencing^28^ can strengthen and provide improved quantification compared to those presented here.

The relative low correspondence between detected peptides (metaproteomics) and active microbial processes compared to the DNA or RNA levels was likely due, at least partially, to the low biomass of nitrifiers and extraction biases due to the complexity of soil matrices as well as limited extraction of membrane proteins, such AmoA, as suggested previously^29^. Alternative proteomic approaches focused on a preselected set of proteins (i.e., selected reaction monitoring or target proteomics) could be used to explore low abundance nitrification proteins. For instance, targeted proteomic approaches have been used to study proteins in low abundance involved in bioremediation pathways in highly-diverse environmental systems^30^. Therefore, targeted proteomics might offer new opportunities for researchers interested in detecting low-abundance peptides and prediction of process rates in complex samples^31^. Finally, abundant peptides related to C1 dehydrogenases that were detected in our metaproteomic profiles (Table S7) were likely not related to denitrification as they did not respond to the nitrogen amendment and were detected in both control and N-amended incubations, and our microcosms were kept under aerobic (non-denitrifying) conditions. Additionally, the annotations of these soil predicted protein sequences might be limited due to generally low identity matches to experimentally verified dehydrogenases.

## Conclusions

The analyses of different omic levels obtained from the incubations showed a high correspondence between nitrification gene markers/transcripts and nitrification process rates. The gene fragments and transcripts were mostly affiliated to novel nitrifier populations similar to those previously described in field soil metagenomes from the same agricultural site^14^. Therefore, the gene and genome sequences reported here could facilitate future investigations of nitrogen cycling in agricultural fields; for instance, by applying qPCR assay targeting the key taxa and biomarker genes and transcripts. The combination of metagenomic and metatranscriptomic approaches used in our study provided a promising strategy for examining microbial activity in agricultural soil environments. Therefore, the findings presented here highlighted the potential of omics data to serve as reliable proxies for examining microbial processes *in situ*, especially in soils, which has been proven to be among the most challenging tasks for environmental studies.

## Methods

### Soil sampling

Our study was focused on an agricultural plot located in the Havana County, Illinois, USA (lat 40.296, long 89.944; elevation, 150 m). The site is representative of the US Midwest and has a long history of conventionally managed corn and soybean crop rotation. In October 2014, we collected ~2 kg of bulk soil from a 20–30 cm soil depth as previous results have shown significant presence of ammonia-oxidizing microorganisms in this layer^14^.

### Soil incubations, gas and chemical analyses

Soil microcosms were established in triplicate, using ~120 g of soil (~8% moisture content) in 500 ml gas-tight canning jars equipped with gas sampling ports, and were sampled at six time points (0, 10, 24, 48, 120, and 192 hours). To simulate a fertilization event in microcosms, 6 ml of 40 mM NH_4_Cl and 20 mM urea (80 mM N) in irrigation water was added to two separate batches of 400 g of soil (Final concentration = 1.2 µmoles-N/g or 18.3 µg-N/g dry weight). Gross rates of nitrification stimulated by the NH_4_^+^ versus the urea fertilizer were estimated using the ^15^N tracer approach with two separate stable isotope treatments (n = 6 per treatment): a 50% ^15^N-NH_4_Cl and 50% ^14^N-NH_4_Cl, 99.7% ^14^N-NH_2_CONH_2_ treatment versus a 50% ^15^N-NH_2_CONH_2_ and 50% ^14^N- NH_2_CONH_2_, 100% ^14^N-NH_4_-Cl treatment. After vigorously mixing, 120 g were dispensed into three separate microcosm jars and incubated in a dark growth chamber with diurnal temperature fluctuation of 22–24 °C as observed in Havana field soil at 20–30 cm during the spring fertilization period (early June). Triplicate microcosms each receiving 6 ml of filtered irrigation water (no nitrogen amendment) served as controls. Individual incubations (whole jar) were sacrificed at each sampling point and all soil (~120 gr) was used for chemical, nucleic acid and protein analyses. After each sampling point, headspace gas was collected from closed jars and the N_2_O concentration was measured on a Shimadzu GC-2014 gas chromatograph (Columbia, MD) equipped with an electron capture detector. Jars were opened for soil sampling and to reestablish equilibration with atmospheric air before being resealed until the next sampling. Ammonium and nitrate in soil subsamples (20 g) were extracted in 2 M KCL and the concentrations were determined using colorimetric analysis on a flow injection auto-analyzer (Lachat Instruments, Milwaukee, WI)^32^. Soil pH (1:1 in water) and gravimetric water content were measured at each time point (Table S1). ^15^N isotopic composition of N_2_O in collected jar headspace samples was determined using an IsoPrime 100 isotope ratio mass spectrometer interfaced with an IsoPrime trace gas analyzer (Cheadle Hulme, UK) at the University of Illinois at Urbana-Champaign. The ^15^N atom % enrichment of the NO_3_^−^ pool was determined using acid trap diffusion^33^ and analysis of the diffusion disks on a Vario Micro Cube elemental analyzer (Elementar, Hanau, Germany) interfaced to an IsoPrime 100 continuous flow isotope ratio mass spectrometer (Cheadle Hulme, UK). ^15^NO_3_^−^ and ^15^N_2_O production rates were calculated from the change in ^15^NO_3_^−^ and ^15^N_2_O concentrations, respectively, from one time point to the following sampling time point. NO_3_^−^ and N_2_O production rates were estimated from the ^15^NO_3_^−^ and ^15^N_2_O production rates based on the mean ^15^N excess atom % of the NH_4_^+^ source pool as described before for N_2_O production rates^34^. No inhibitors of nitrogen cycle pathways were used in the incubations.

### Nucleic acid extractions

DNA and RNA were extracted independently but from the same soil of each incubation at each time point. DNA was extracted from ~0.5 g of soil from individual incubations (i.e., replicates were not mixed) using a modified phenol-chloroform and purification protocol as previously described^35^. For RNA extraction, 2 gr of soil was preserved in LifeGuard (MoBio) and stored at − 80 °C. A modified protocol derived from the PowerMax Soil DNA kit for extracting RNA was used for total RNA extractions (MoBio). TURBO DNAse (Ambion) was used to remove DNA according to the recommendations of the manufacturer. Nucleic acid extracts were quantified using Quant-it ds DNA HS and HS RNA assays (Invitrogen) according to the instructions of the manufacturer. RNA quality was assessed using Agilent RNA 6000 pico kit (Agilent Technologies) and samples having RNA integrity number (RIN) above 7 were used. Additional details about nucleic acid sequencing are available in Supplementary Material.

### Short-read analyses

Metagenomic and metatranscriptomic raw reads (FASTQ) for sequenced samples were trimmed using SolexaQA^36^ using a Phred score cutoff of 20 and minimum fragment length of 50 bp. Short-reads derived from metatranscriptomes were merged using PEAR using default parameters^37^. Average coverage for each sequenced metagenome was determined by Nonpareil^38^ using default settings except that 2,000 reads were used as query (-X option). Short-read sequences encoding 16S rRNA gene fragments were extracted from each metagenome and metatranscriptome by SortMeRNA^39^ and their taxonomy was assigned using RDP classifier (cutoff 50)^40^.

To identify and quantify reads encoding specific protein sequences of interest, we used the previously published protein sequences as references^14,41^. Independent ROCker^18^ models (length = 125 bp) were subsequently built based on these reference protein sequences with the exception of NarG and NxrA, where the sequences were combined into a single model. Trimmed short-reads from soil metagenomes were used as query for BLASTx searches (e-value 0.01) against the latter protein databases and outputs were filtered using the previously generated ROCker models. For metagenomes, target gene abundance in metagenomes was determined as genome equivalents by calculating the ratio between normalized target reads (number of reads matching divided by median protein length) and normalized RpoB reads (number of reads matching divided by median RpoB protein length), a universal single-copy gene. For metatranscriptomes, target transcripts abundance was calculated as reads per kilobase of transcript per million mapped reads (RPKM). Protein databases and ROCker models are available through http://enve-omics.ce.gatech.edu/.

### Assembly and binning of metagenomic populations

Short-read metagenomes from control and treatments (t = 0,120 and 192 hours) were co-assembled using IDBA_UD v1.1.1^42^ and binning was performed as previously described^14^. Taxonomic classification and degree of novelty (novel species, genus, etc) of the MAGs were obtained from the Microbial Genomes Atlas (MiGA) webserver^43^. MAG abundance was determined as the total length of all matching metagenomic or metatranscriptomic reads to the binned contigs from BLASTn searches (identity >= 98% and fraction of read aligned >= 50%) divided by the metagenomic or metatranscriptomic sample sizes (in millions of reads) and the length of the bin genomes in Kbp (Kilo base pairs). Reads encoding rRNA sequences (such as 5S, 5.8S, 16S, and 23S) were identified by SortMeRNA, and removed for non-rRNA analyses in order to avoid overestimating abundances.

N cycle protein sequences in the co-assembly and MAGs were detected using hidden Markov models obtained from FUNGENE^44^, using HMMer^45^. Detected target N cycle proteins were manually curated, when necessary, by assessing the presence of characteristic amino acid and phylogenetic congruency.

### Phylogenetic trees and placement of short-reads

To assess the phylogenetic affiliation of metagenomic or metatranscriptomic reads, reference and fully assembled protein sequences were aligned using ClustalΩ^46^ with default parameters. Resulting alignments were used to build phylogenetic trees in RAxML v8.0.19^47^. Short*-*reads encoding the protein of interest were extracted from metagenomes or metatranscriptomes using ROCker (BLASTx) and placed in their corresponding phylogenetic tree using the methodology previously described^14^. Quantification of the number of reads assigned to a specific clade (e.g., to distinguish between *nxrA* or *narG* reads) was done using the “JPlace.distances.rb” script, also available in the enveomics collection. To quantify *nirK* gene fragments assigned to specific clades, the same process as described above was repeated except that all reads detected by multiple ROCker models to previously described clades^48^ (clades I + II, III and *Thaumarchaeotea*) were used. Abundances of target genes were determined as reads per kilobase million (RPKM) for metatranscriptomics. Equivalent results were obtained when using transcripts per kilobase million (TPM) but RPKM values were preferred for our targeted approach that focused on a reduced set of gene transcripts (*ureC*, *amoA*, *haoA*, *nxrA*, and *nirK*) and nitrifier populations.

### Shotgun metaproteomics

Approximately 10 g of soil were collected from the 192 hours control and ^15^N-NH_4_^+^ amended microcosms and stored at − 80 °C. Frozen soil (5 g) was used for protein extractions. A detailed procedure is available in the Supplementary Material.

### NanoLC-MS/MS analysis

Peptides (75 ug) were loaded onto in-house prepared biphasic resin packed column [SCX (Luna, Phenomenex, Torrance, CA) and C18 (Aqua, Phenomenex, Torrance, CA)] as described earlier^49,50^ and subjected to an offline wash for 15 min as previously described^51^. The sample column was aligned with an in-house C18 packed nanospray tip (New Objective, Woburn, MA) connected to a Proxeon (Odense, Denmark) nanospray source as previously detailed^51^. Peptides were eluted and subjected to chromatographic separation and measurements via 24-hr Multi-Dimensional Protein Identification Technology (MuDPIT) approach as described earlier^49–51^. Measurements were carried out using LTQ mass spectrometer (Thermo Fisher Scientific, Germany) coupled to the Ultimate 3000 HPLC system (Dionex, USA) and operated in data dependent mode, via Thermo Xcalibur software V2.1.0 as described earlier^49^.

For protein identification, the raw spectra from each run were searched against a custom database and was constructed using protein sequences predicted from metagenome assemblies obtained from the same soil and 20–30 cm depth^14^, metagenome assemblies from incubations (Table S2), and reference proteomes for 47 common soil organisms (Table S6). Detected proteins predicted from metagenomic assemblies were annotated using BLASTp^52^ and UniProt database as reference^53^ (downloaded in May of 2017). Additional details are available in the Supplementary Material.

## Supplementary information

Supplementary material

## Data Availability

Raw metagenomic and metatranscriptomic soil datasets and MAGs are deposited in the European Nucleotide archive (ENA) under study number PRJEB27434. MAGs previously recovered from the same agricultural site^14^ are deposited in ENA under study number PRJEB20068 and are also available at http://enve-omics.ce.gatech.edu/data/.
