# Supplementary material for: Comparing DNA, RNA and protein levels for measuring microbial dynamics in soil microcosms amended with nitrogen fertilizer
Source: Sci Rep. 2019 Nov 26;9:17630. doi: 10.1038/s41598-019-53679-0 (PMC6879594; doi:10.1038/s41598-019-53679-0)
Supplement: Supplementary file 1 — Supplementary material [file 41598_2019_53679_MOESM1_ESM.pdf]

Comparing DNA, RNA and protein levels for measuring microbial dynamics in soil microcosms amended with nitrogen fertilizer

Luis H. Orellana,[a] Janet K. Hatt,[a] Ramsunder Iyer,[b,c] Karuna Chourey,[b] Robert L. Hettich,[b] Jim C. Spain,[d] Wendy H. Yang,[e] Joanne C. Chee-Sanford,[f] Robert A. Sanford,[g] Frank E. Löffler,[h,i] Konstantinos T. Konstantinidis[a,#]

[a] School of Civil and Environmental Engineering, Georgia Institute of Technology, Atlanta, Georgia, USA
[b] Chemical Sciences Division, Oak Ridge National Laboratory, Oak Ridge, Tennessee, USA
[c] Graduate School of Genome Science and Technology, University of Tennessee, Knoxville, Tennessee, USA
[d] Center for Environmental Diagnostics & Bioremediation, University of West Florida, Pensacola, Florida, USA
[e] Department of Geology, University of Illinois at Urbana-Champaign, Urbana, Illinois USA
[f] U.S. Department of Agriculture, Agricultural Research Service, Urbana, Illinois, USA
[g] University of Illinois at Urbana-Champaign, Urbana, Illinois, USA
[h] University of Tennessee, Knoxville, Tennessee, USA
[i] Biosciences Division, Oak Ridge National Laboratory, Oak Ridge, Tennessee, USA

Supplementary Results

Soil Metagenomes and Metatranscriptomes

To explore the genomic potential of microbial communities in control and nitrogen-amended microcosms, we examined the metagenomes and metatranscriptomes obtained from the incubated soils. Metagenomes ranged from 23.7 to 53.4 million short-reads and metatranscriptomes from 10.1 to 31.3 million short-reads per sample (Tables S2 and S3). The estimated average coverage based on read redundancy using Nonpareil[1], i.e., the fraction of the total extracted DNA that was sequenced, ranged from 0.27 to 0.42 for the soil metagenomes (values range from 0 to 1). The co-assembly of selected soil metagenomes generated 1.52 million contigs over 500 bp (assembly N50=1,176) and 1.56 million predicted protein-coding genes.

A high fraction of ribosomal RNA was detected for all metatranscriptomes ranging from 94% to 98% of the total sequences (Table S4). No rRNA depletion step was performed during our metatranscriptomic protocol due to overall low total RNA yields from the soils. As expected based

on the length of the rRNA genes, 23S rRNA/16S rRNA ratios ranged from 1.7 to 1.9, indicating adequate RNA quality. Bacterial 16S rRNA was the most abundant, ranging between 30.6% and 35.9% of total transcripts per sample. Archaeal 16S rRNA and eukaryotic 18S rRNA molecules were less abundant, with values ranging from 0.09% to 0.15% and 0.55 to 2.9%, respectively. The estimated average coverage for the metatranscriptomic datasets –after removing rRNA-encoding reads- ranged from 0.77 to 0.84 (avg = 0.81). These coverage values reflected an adequate representation of microbial community gene transcripts in our datasets. Note, however, that the coverage values obtained between metatranscriptomes and metagenomes may not be directly comparable due to uneven individual gene abundance (because of differential transcription activity) in addition to genome abundance and underrepresentation of intergenic regions in the former dataset. The annotation of the transcripts revealed that the most abundant annotated functions reached the highest relative expression at 48h of incubation (Fig. S6). Functions related to protein and RNA metabolism, among others, represented the most abundant transcripts detected throughout the incubations and had higher abundances during the first two days. Other functions related to motility and chemotaxis, metabolism of aromatic compounds and central metabolisms were less abundant but stably expressed during the incubation. In agreement with proteomic results, similar highly abundant functional categories such as those related to protein and RNA metabolism were also observed at 192 h of incubation (Figure S6).

Taxonomy of microbial soil populations based on 16S rRNA gene sequences

The taxonomic composition and abundances of the main microbial groups were determined from recovered 16S rRNA gene sequences (DNA level) from nitrogen-amended incubations. Relative abundances of taxa were determined by dividing the number of their 16S rRNA reads with the total number of 16S rRNA reads detected by SortMeRNA. At the class taxonomic level, *Actinobacteria*, *Betaproteobacteria*, and *Gammaproteobacteria* were the most abundant groups in metagenomes, accounting for more than 57% of the total community in

nitrogen-amended incubations (Fig. S2). The taxonomic composition derived from metatranscriptomes (cDNA reads) was also stable during the incubations but the relative abundance patterns for the main taxonomic groups were substantially different from the metagenomes. For instance, *Betaproteobacteria*, *Gammaproteobacteria*, and *Flavobacteria* were among the most abundant groups in cDNA samples, accounting for an average of 77.5% of the 16S rRNA gene transcripts.

Individual populations from microcosms metagenomes

The assembly and binning of the soil metagenomes recovered 11 metagenome-assembled genomes (MAGs) mostly representing *Proteobacteria*, *Acidobacteria*, *Actinobacteria* and *Nitrospirae* phyla. Most of the recovered MAGs represented novel genera (n=7) and species (n=5) when the taxonomic novelty was evaluated against 10,487 reference genomes (taxonomically classified at the species level) using genome-aggregate amino acid identity (AAI) thresholds for taxonomic rank delineation[2] (Table S5). The fraction of total metagenomic reads mapping to these MAGs ranged from 4.1% to 5.7% depending on the dataset considered, with an average of 4.8% (threshold for mapping: identity ≥ 95% and fraction of read aligned >70%). These seven MAGs (from previous metagenomic project) recruited between 0.36% to 0.77% of the metagenomic reads in each dataset with an average of 0.55%, representing a smaller fraction of the metagenomes compared to the non-nitrifier MAGs described above. Similarly, the MAGs recovered from the incubations and field recruited, on average, 0.92% and 0.46% of the transcriptomic libraries.

Supplementary Methods

Nucleic Acid Sequencing

For metagenomes, dual-indexed DNA sequencing libraries were prepared using the Illumina Nextera XT DNA library prep kit according to manufacturer's instructions, except that the

protocol was terminated after isolation of cleaned amplified double stranded libraries. For metatranscriptomes, single-indexed cDNA sequencing libraries were prepared using ScriptSeq v2 protocol using ~25 ng of total RNA as input. All DNA and cDNA library concentrations were determined by fluorescent quantification using a Qubit HS DNA kit and Qubit 2.0 fluorometer (ThermoFisher Scientific) according to manufacturer's instructions and samples were run on a High Sensitivity DNA chip using the Bioanalyzer 2100 instrument (Agilent) to determine quality and average library insert sizes. An equimolar mixture of the libraries was sequenced on an Illumina HiSEQ 2500 instrument (School of Biological Sciences, Georgia Institute of Technology) for a rapid run of 300 cycles (2 x 150 bp paired end) using the HiSeq Rapid PE Cluster Kit v2 and HiSeq Rapid SBS Kit v2 (Illumina). Adapter trimming and demultiplexing of sequenced samples was carried out by the Illumina software, according to the recommendations of the manufacturer.

Shotgun Metaproteomics

Frozen soil (5 g) was thawed and suspended in lysis buffer and boiled for 15 minutes as described previously[3].The supernatant was retained and amended with 100% chilled TCA to final concentration of 25% (vol/vol) and kept at -20°C overnight. Samples were centrifuged at 21,000 x *g* for 20 min and the protein pellets processed as described previously[4] and solubilized in 6 M guanidine buffer (6 M guanidine; 10 mM dithiothreitol [DTT] in Tris-$CaCl_2$ buffer (10 mM Tris; , pH 7.8) with 3 hr incubation at 60°C. An aliquot of 25 µl/ sample was retained for protein estimation and the rest of the protein sample was digested, peptides desalted and solvent exchanged as described earlier[5]. The amount of protein extracted from each sample was calculated using the RC/DC protein estimation kit (Bio-Rad Laboratories, Hercules, CA, USA) as per the manufacturer's instructions. Bovine serum albumin (supplied with the kit) was used as standard for the assay. All chemicals were obtained from Sigma Chemical Co. (St. Louis, MO), unless specified otherwise. High performance liquid chromatography- (HPLC-) grade water and other solvents were obtained from Burdick & Jackson (Muskegon, MI), 99% formic acid was purchased

from EM Science (Darmstadt, Germany) and sequencing-grade trypsin was acquired from Promega (Madison, WI).

Identification and annotation of raw spectra

The predicted proteins from metagenomes and refence proteomes were used for constructing a database for metaproteomic searches (available through http://enve-omics.ce.gatech.edu/data/multiomics-soil). Database matching was done via Myrimatch v2.1 algorithm[6] set to parameters described before[7] with minor modifications where static cysteine and dynamic oxidation modifications were not considered. Identification of at least two peptides per protein (one unique and one non-unique) sequence was a prerequisite for protein identifications. Common contaminant peptide sequences from trypsin and keratin were concatenated to the database. Reverse database sequences were also included in the database as decoy sequences to calculate false discovery rate (FDR). For data analysis, spectral counts of identified peptides was normalized as described before[8] to obtain the normalized spectral abundance factor (NSAF) and the NSAF values were multiplied by a constant number (100,000) for better visualization and referred to as normalized spectral counts (nSpc). The nSpc were used to compare expression of proteins across different samples and different time points.

Legends for supplementary figures and tables

Supplementary Figure 1. Mean nitrification rates and N_2O for ^{15}N-NH_4 and ^{15}N-urea microcosms. Error bars represent the standard deviation (n=3).

Supplementary Figure 2. Taxonomic composition of soil incubations. Summary of the taxonomic affiliation of recovered 16S rRNA gene and transcript fragments from metagenomes and metatranscriptomes.

Supplementary Figure 3. Relative expression ratio (cDNA/DNA) for 16S rRNA gene fragments related to nitrifier communities (genus) level. Expression ratios were determined using 16S rRNA gene fragments extracted from metagenomes and metatranscriptomes from soil microcosms amended with $^{15}N\text{-}NH_4Cl$ and $^{14}N\text{-}NH_4Cl$.

Supplementary Figure 4. Relative abundance of indigenous ammonia-oxidizing archaea, ammonia-oxidizing bacteria and nitrite-oxidizing bacteria population genomes in metatranscriptomes. A. Relative expression values (RPKM) values for nitrifier MAGs obtained from incubation metagenomes and previously sequenced field metagenomes are shown for each incubation point. B. MAGs were queried for the presence of nitrification gene markers using HMM models. Each shape represents an individual MAG. Arrows show predicted nitrogen cycle pathways. C. Variability between populations from field and incubation metagenomes was determined by mapping the short-reads from different metagenomes to MAGs using BLASTn. The average nucleotide identity of the mapped reads (ANIr) was determined for the MAGs in all samples.

Supplementary Figure 5. Nitrification genes in incubated soils. A. Relative expression ratios (cDNA) for each nitrification step in incubated soils were determined at 10, 48, 120, and 192 hours incubation. Panel B shows relative abundances at the DNA level (RPKM) at 120 and 192 hours of incubation. Panel C shows the relationship between measured bacterial *amoA* transcripts and nitrate accumulation (quadratic and linear regressions).

Supplementary Figure 6. List of the most abundant SEED subsystems in metatranscriptomes. All non-rRNA transcripts obtained at 10, 48, 120, and 192 hours of incubation were annotated using UniProt and summarized using the hierarchical approach of

the SEED subsystems. Non-specific subsystems such as “Miscellaneous” and “Clustering-based subsystems” were excluded from the figure.

Supplementary Table 1. pH of incubated soils.

Supplementary Table 2. Soil incubation metagenome statistics.

Supplementary Table 3. Soil incubation metatranscriptome statistics.

Supplementary Table 4. Ribosomal gene fragments recovered from metagenomes and metatranscriptomes.

Supplementary Table 5. Statistics and taxonomy and statistics for recovered bins (MiGA results).

Supplementary Table 6. Reference soil microorganisms used as part of the database for spectra annotation.

Supplementary Table 7. Annotations for most abundant detected peptides in control and N-amended incubations.

References

1. Rodriguez-R, L. M. & Konstantinidis, K. T. Nonpareil: a redundancy-based approach to assess the level of coverage in metagenomic datasets. *Bioinformatics* **30,** 629–635 (2014).
2. Luo, C., Rodriguez-R, L. M. & Konstantinidis, K. T. MyTaxa: an advanced taxonomic classifier for genomic and metagenomic sequences. *Nucleic Acids Res.* **42,** e73–e73 (2014).
3. Chourey, K. *et al.* Direct cellular lysis/protein extraction protocol for soil metaproteomics. *J. Proteome Res.* **9,** 6615–6622 (2010).
4. Chourey, K. *et al.* Environmental proteomics reveals early microbial community responses to biostimulation at a uranium- and nitrate-contaminated site. *Proteomics* **13,** 2921–2930 (2013).
5. Thompson, M. R. *et al.* Dosage-dependent proteome response of *Shewanella oneidensis* MR-1 to acute chromate challenge. *J. Proteome Res.* **6,** 1745–1757 (2007).
6. Tabb, D. L., Fernando, C. G. & Chambers, M. C. MyriMatch: highly accurate tandem mass spectral peptide identification by multivariate hypergeometric analysis. *J. Proteome Res.* **6,** 654–661 (2007).
7. Xiong, W., Giannone, R. J., Morowitz, M. J., Banfield, J. F. & Hettich, R. L. Development of an enhanced metaproteomic approach for deepening the microbiome characterization of the human infant gut. *J. Proteome Res.* **14,** 133–141 (2015).
8. Paoletti, A. C. *et al.* Quantitative proteomic analysis of distinct mammalian Mediator complexes using normalized spectral abundance factors. *Proc Natl Acad Sci USA* **103,** 18928–18933 (2006).

Supplementary Figure 1

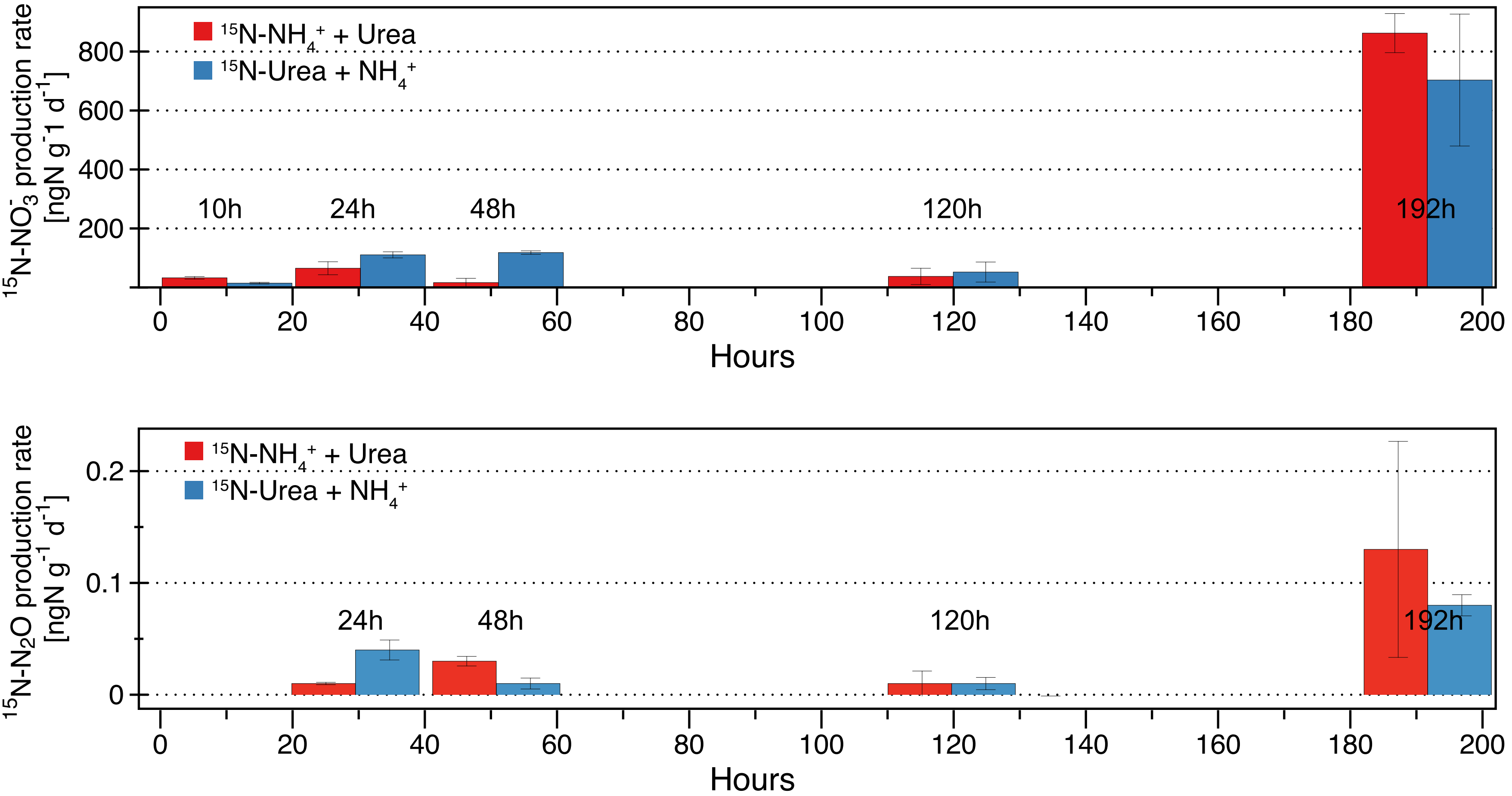

Supplementary Figure 2

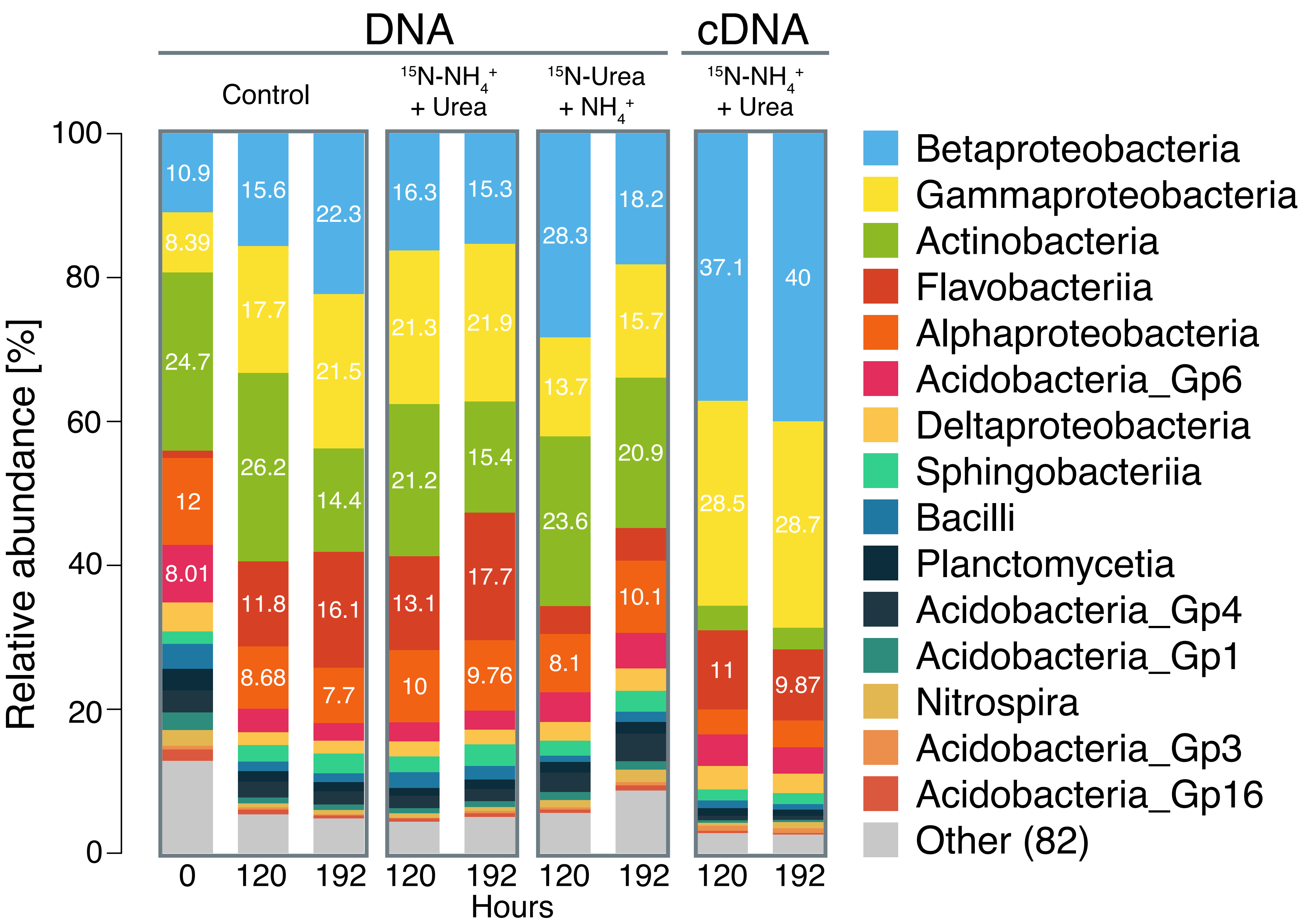

Supplementary Figure 3

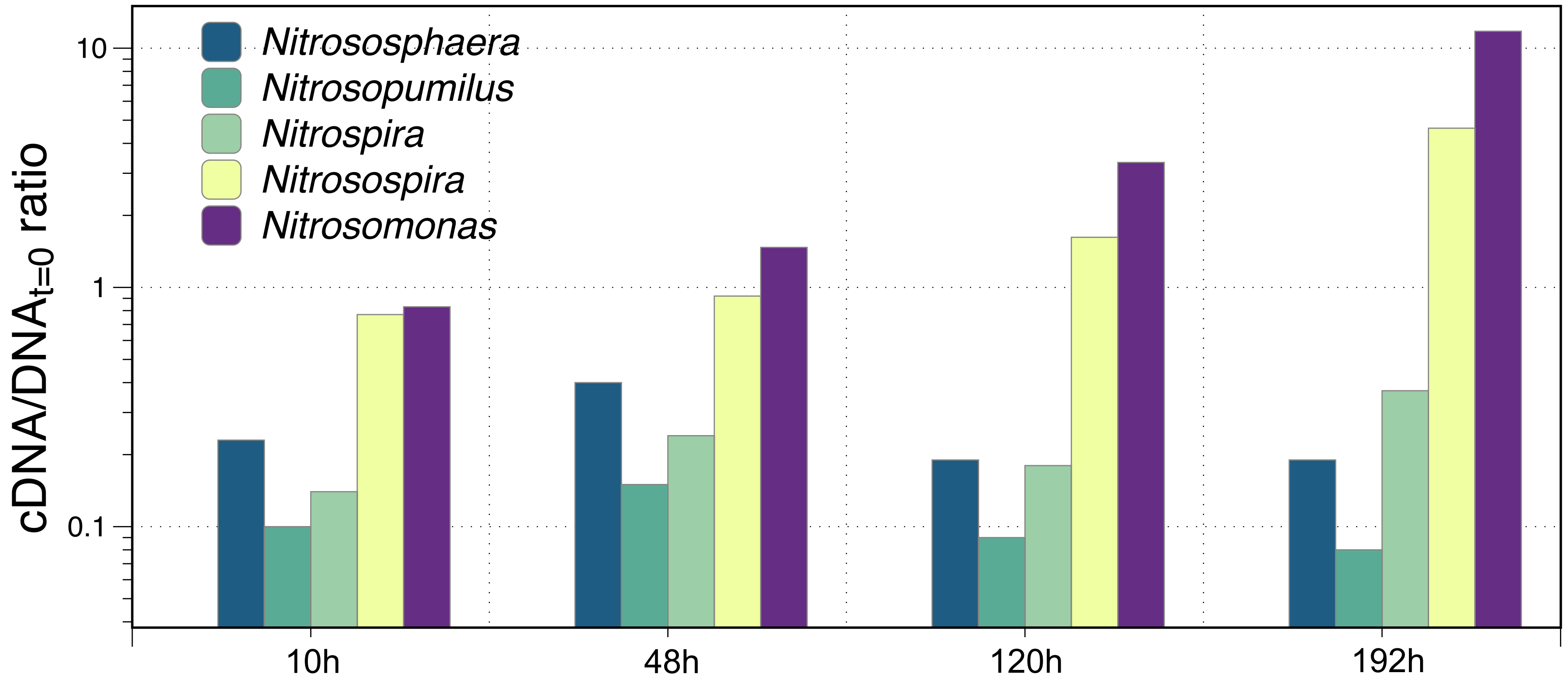

Supplementary Figure 4

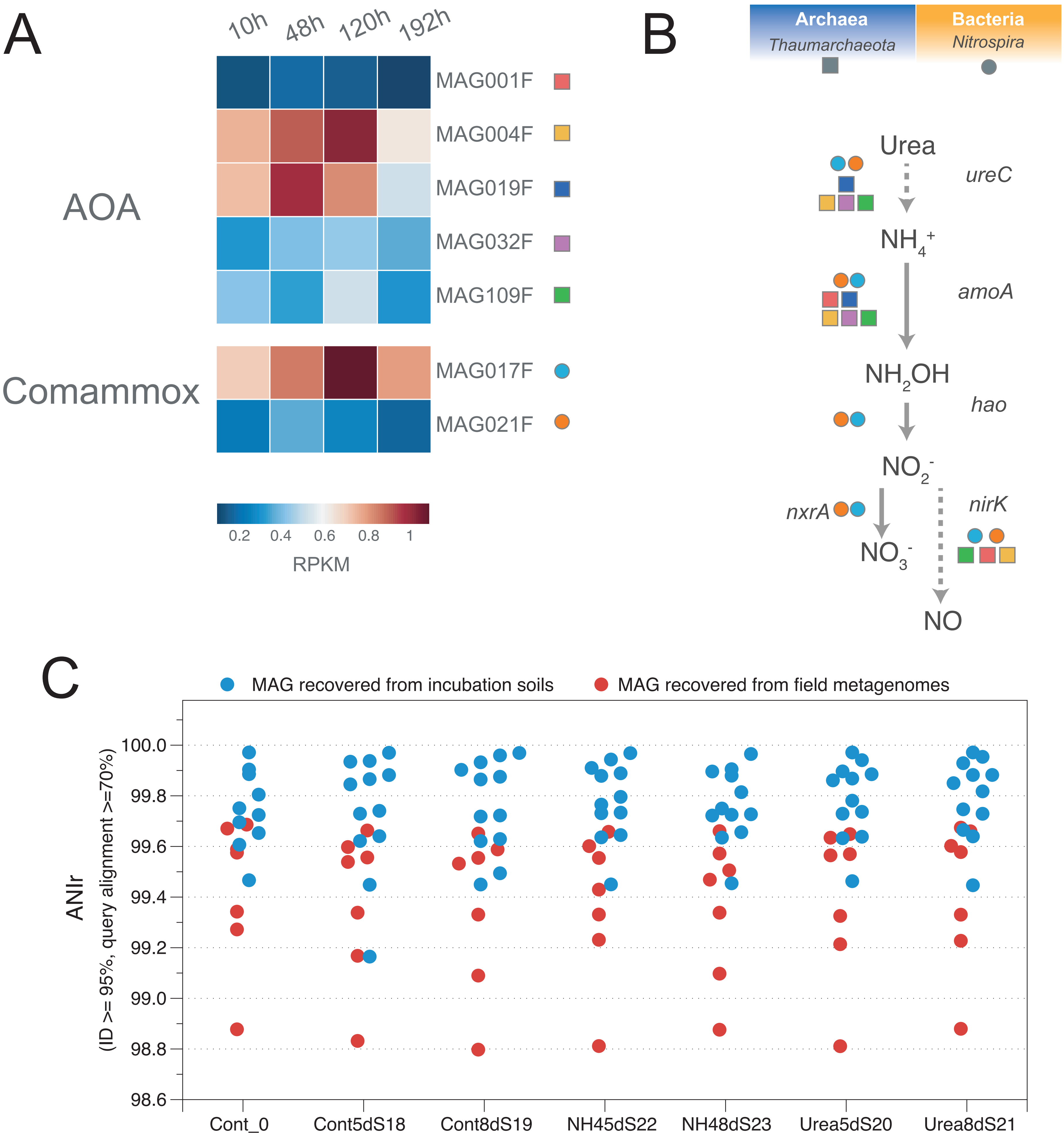

Supplementary Figure 5

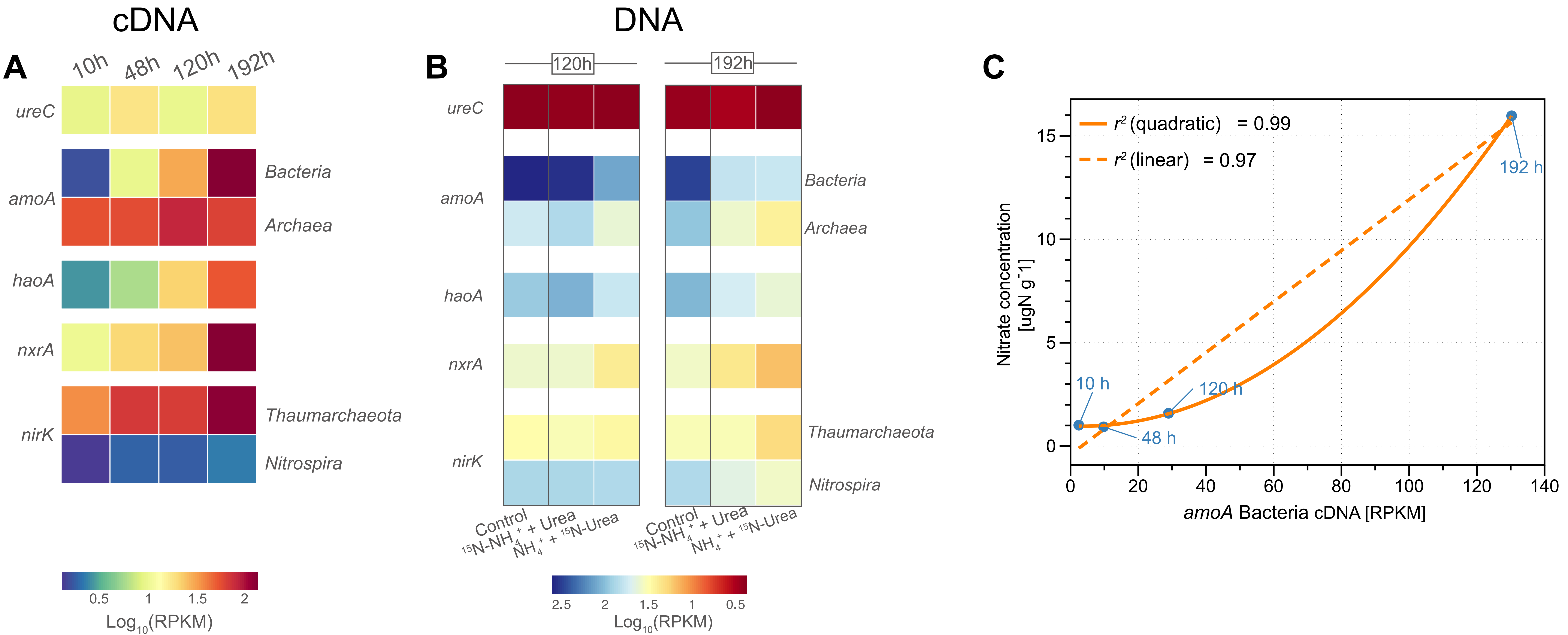

Supplementary Figure 6

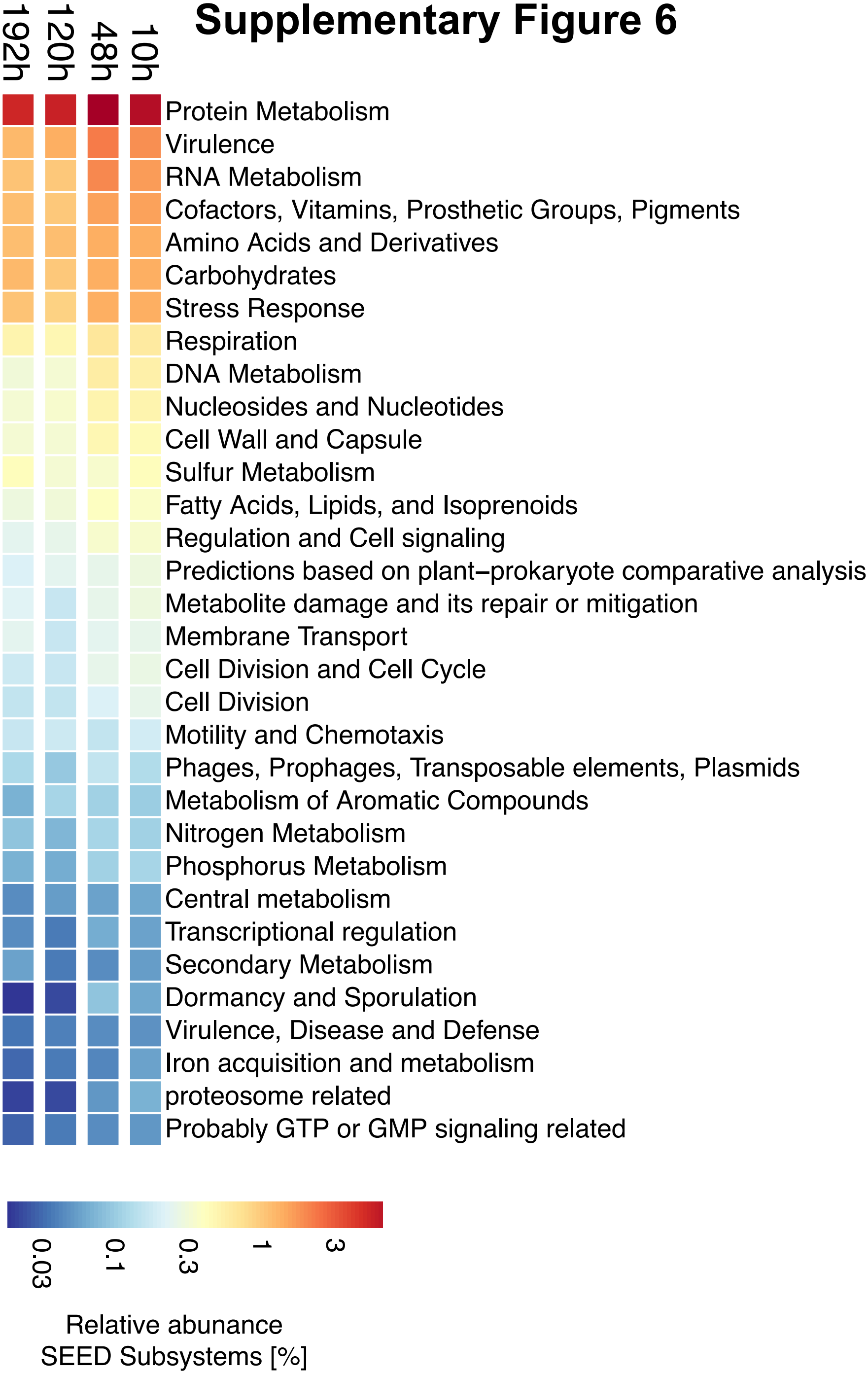

Supplementary Table 1

Treatment	Time [h]	pH
Control	0	7.42 ± 0.09
	10	7.51 ± 0.06
	24	7.49 ± 0.06
	48	7.38 ± 0.23
	120	7.48 ± 0.05
	192	7.54 ± 0.03
^{15}N-NH_4^+ + Urea	0	7.53 ± 0.02
	10	7.61 ± 0.03
	24	7.64 ± 0.05
	48	7.59 ± 0.02
	120	7.50 ± 0.04
	192	6.60 ± 0.13
^{15}N-Urea + NH_4^+	0	7.34 ± 0.10
	10	7.52 ± 0.02
	24	7.62 ± 0.03
	48	7.56 ± 0.03
	120	7.42 ± 0.07
	192	6.78 ± 0.07

Supplementary Table 2

ID	Test	Time [h]	Trimming		Nonpareil
			Coupled reads	Avg. read length	Coverage
Cont_0	Control	0	53,481,680	140.9	0.424
Cont5dS18		120	23,780,198	140.1	0.308
Cont8dS19		192	32,122,126	141.8	0.369
NH45dS22	^{15}N-NH_4^+ + Urea	120	24,204,426	141.5	0.294
NH48dS23		192	36,660,412	139.9	0.371
Urea5dS20	^{15}N-Urea + NH_4^+	120	28,976,856	141.6	0.356
Urea8dS21		192	32,139,060	142.3	0.274

Supplementary Table 3

ID	Treatment	Time [h]	Single reads after merging			Not merged reads	
			Merged reads	Percentage merged	Avg. read length	Not merged reads	Avg. read length
Amo_10h	^{15}N-NH_4^+ + Urea	10	23,252,114	79.0	154.3	8,101,024	140.7
Amo_48h		48	23,628,975	80.3	150.9	7,233,008	140.9
Amo_5d		120	8,305,590	85.0	143.5	1,829,636	140.1
Amo_8d		192	13,392,531	76.3	152.2	5,789,762	139.7

Supplementary Table 4

Source	ID	rRNA										Non rRNA
		Bacterial 16S	Archaea 16S	5.8S	5S	Achaea 23S	Bacteria 23S	Eukarya 18S	Eukarya 28S	16S/23S ratio	Total [%]	Non RNA [%]
DNA	Cont_0	0.06	< 0.01	< 0.01	0.01	< 0.01	0.12	0.01	0.01	0.50	0.20	99.8
	Cont5dS18	0.08	< 0.01	< 0.01	0.01	< 0.01	0.17	< 0.01	< 0.01	0.47	0.26	99.7
	Cont8dS19	0.09	< 0.01	< 0.01	0.01	< 0.01	0.18	< 0.01	0.01	0.50	0.29	99.7
	NH45dS22	0.08	< 0.01	< 0.01	0.01	< 0.01	0.16	< 0.01	< 0.01	0.50	0.25	99.7
	NH48dS23	0.09	< 0.01	< 0.01	0.01	< 0.01	0.17	< 0.01	0.01	0.53	0.27	99.7
	Urea5dS20	0.08	< 0.01	< 0.01	0.01	< 0.01	0.16	< 0.01	< 0.01	0.50	0.25	99.7
	Urea8dS21	0.07	< 0.01	< 0.01	0.01	< 0.01	0.14	< 0.01	0.01	0.50	0.23	99.8
cDNA	Amo_10h	30.76	0.1	0.1	0.04	0.22	57.89	2.06	3.96	95.13	95.13	4.9
	Amo_48h	30.59	0.15	0.14	0.05	0.35	55.63	2.9	4.81	94.62	94.62	5.4
	Amo_5d	35.91	0.09	0.07	0	0.22	60.37	0.55	0.72	97.93	97.93	2.1
	Amo_8d	33.21	0.09	0.07	0	0.24	62.85	0.71	0.95	98.12	98.12	1.9

Supplementary Table 5

	Total length [bp]	G+C content [%]	Contigs	Predicted proteins	Taxonomic classification	Taxonomic novelty	Complet.	Cont.	Closest relative
**MAG_001**	2,795,863	66.89	593	3,282	**phylum** *Actinobacteria* (p-value: 0.003)	genus (p-value: 0)	74.8%	1.8%	*Rubrobacter xylanophilus* DSM 9941 (41.31% AAI)
**MAG_004**	5,929,205	55.16	112	5,100	**phylum** *Acidobacteria* (p-value: 1.7e-05)	genus (p-value: 0.0018)	93.7%	2.7%	*Chloracidobacterium thermophilum* B (42.78% AAI)
**MAG_006**	6,545,070	57.99	382	6,197	**class** *Acidobacteriia* (p-value: 0)	species (p-value: 0.0031)	90.1%	2.7%	*Candidatus* Koribacter versatilis Ellin345(53.5% AAI)
**MAG_007**	4,447,407	55.55	697	5,213	**family** *Nitrospiraceae* (p-value: 0.0023)	species (p-value: 0.0038)	88.3%	0.9%	*Nitrospira japonica* (60.97% AAI)
**MAG_012**	6,020,047	55.96	1,089	5,892	**phylum** *Acidobacteria* (p-value: 0)	genus (p-value: 0.0018)	70.3%	0.9%	*Candidatus* Solibacter usitatus Ellin6076 (42.81% AAI)
**MAG_015**	4,264,353	67.13	699	4,060	**phylum** *Acidobacteria* (p-value: 0.00048)	genus (p-value: 0)	77.5%	1.8%	*Candidatus* Solibacter usitatus Ellin6076 (41.97% AAI)
**MAG_021**	5,335,416	64.73	770	5,679	**phylum** *Gemmatimonadetes* (p-value: 0)	genus (p-value: 0.0029)	79.3%	4.5%	*Gemmatimonas phototrophica* (44.96% AAI)
**MAG_031**	4,140,833	58.05	438	3,852	**phylum** *Acidobacteria* (p-value: 0)	genus (p-value: 0.0021)	88.3%	0.0%	*Candidatus* Solibacter usitatus Ellin6076 (43.3% AAI)
**MAG_144**	2,739,318	69.89	244	2,913	**order** *Propionibacteriales* (p-value: 0.0096)	species (p-value: 0.0038)	62.2%	2.7%	*Nocardioides* sp. 78 (57.05% AAI)
**MAG_167**	1,707,495	53.49	430	1,973	**class** *Gammaproteobacteria* (p-value: 0)	species (p-value: 0.0038)	66.7%	0.9%	*Lysobacter capsici* (55.73% AAI)
**MAG_176**	3,660,466	60.72	240	3,620	**family** *Oxalobacteraceae* (p-value: 0)	species (p-value: 0.0062)	89.2%	1.8%	*Herbaspirillum* sp. meg3 (66.29% AAI)
**MAG_178**	4,531,074	66.02	105	4,132	**class** *Gammaproteobacteria* (p-value: 0.0063)	genus (p-value: 0.0055)	91.9%	0.9%	*Thiohalobacter thiocyanaticus* (45.73% AAI)

Supplementary Table 6

Reference proteome
Anaeromyxobacter dehalogenans 2CP-1
Anaeromyxobacter sp. Fw109-5
Gemmatimonas aurantiaca
Gemmatirosa kalamazoonensis
Bradyrhizobium japonicum USDA
Dyadobacter fermentans
Nitrososphaera gargensis (strain Ga9.2)
Candidatus Nitrosopumilus sp. AR2
Candidatus Nitrosoarchaeum koreensis MY1
Candidatus Nitrospira inopinata
Nitrosomonas europaea
Nitrospira moscoviensis
Candidatus Nitrospira nitrosa
Conexibacter woesei (strain DSM 14684 / JCM 11494 / NBRC 100937 / ID131577)
Nocardioides sp. (strain BAA-499 / JS614)
Pseudogulbenkiania sp. (strain NH8B)
Rhodoferax ferrireducens (strain ATCC BAA-621 / DSM 15236 / T118)
Acidovorax avenae (strain ATCC 19860 / DSM 7227 / JCM 20985 / NCPPB 1011)
Phenylobacterium zucineum (strain HLK1)
Oligotropha carboxidovorans (strain ATCC 49405 / DSM 1227 / OM5)
Hyphomicrobium denitrificans (strain ATCC 51888 / DSM 1869 / NCIB 11706 / TK 0415)
Ralstonia pickettii (strain 12D)
Leptothrix cholodnii (strain ATCC 51168 / LMG 8142 / SP-6)
Actinosynnema mirum (strain ATCC 29888 / DSM 43827 / NBRC 14064 / IMRU 3971)
Geobacillus kaustophilus (strain HTA426)
Dechloromonas aromatica (strain RCB)
Accumulibacter phosphatis (strain UW-1)
Thioalkalivibrio nitratireducens (strain DSM 14787 / UNIQEM 213 / ALEN2)
Frankia alni (strain ACN14a)
Nocardia farcinica (strain IFM 10152)
Mycobacterium sp. (strain KMS)
Sorangium cellulosum (strain So ce56)
Paracoccus denitrificans (strain Pd 1222)
Thaumarchaeota archaeon N4
Nitrosospira multiformis (strain ATCC 25196 / NCIMB 11849)
Gaiella sp. SCGC AG-212-M14
Flavobacterium johnsoniae
Candidatus Saccharibacteria bacterium GW2011_GWC2_48_9
Aeromicrobium sp. Root344
Pseudomonas fluorescens (strain Pf0-1)
Koribacter versatilis (strain Ellin345)
Acidobacterium capsulatum (strain ATCC 51196 / DSM 11244)
Marmoricola sp. Leaf446
Nocardioides sp. Soil777
Sphingomonas sanxanigenens DSM 19645 = NX02
Solirubrobacter soli
Rhodopseudomonas palustris (strain ATCC BAA-98 / CGA009)

Supplementary Table 7

Incubation	Abundance Ranking	Average SpC	Predicted function	Best match organisms
Control	1	724.6	Chaperone protein DnaK	Methylococcus capsulatus (strain ATCC 33009 / NCIMB 11132 / Bath)
	2	403.63	Elongation factor Tu	Rhodopseudomonas palustris (strain BisB18)
	3	361.75	Flagellin	Leptothrix cholodnii (strain ATCC 51168 / LMG 8142 / SP 6)
	4	329.57	Methanol dehydrogenase large subunit like protein	Bradyrhizobium diazoefficiens (strain JCM 10833 / IAM 13628 / NBRC 14792 / USDA 110)
	5	313.55	ATP dependent Clp protease ATP binding subunit	Gaiella sp. SCGC AG 212 M14
	6	299.14	Bll6026 protein	Bradyrhizobium diazoefficiens (strain JCM 10833 / IAM 13628 / NBRC 14792 / USDA 110)
	7	294.02	Bll6025 protein	Bradyrhizobium diazoefficiens (strain JCM 10833 / IAM 13628 / NBRC 14792 / USDA 110)
	8	285.42	ATP dependent Clp protease ATP binding subunit	Caldanaerobacter subterraneus subsp. tengcongensis (strain DSM 15242 / JCM 11007 /
	9	283.48	Bll6026 protein	Bradyrhizobium diazoefficiens (strain JCM 10833 / IAM 13628 / NBRC 14792 / USDA 110)
	10	283.48	Putative outer membrane protein B, OmpB	Koribacter versatilis (strain Ellin345)
	11	270.87	ATP dependent Clp protease ATP binding subunit	Koribacter versatilis (strain Ellin345)
	12	263.17	Elongation factor Tu	Burkholderia lata (strain ATCC 17760 / LMG 22485 / NCIMB 9086 / R18194 / 383)
	13	239.04	Formaldehyde activating enzyme	Hyphomicrobium denitrificans (strain ATCC 51888 / DSM 1869 / NCIB 11706 / TK 0415)
	14	227.23	Bll6025 protein	Bradyrhizobium diazoefficiens (strain JCM 10833 / IAM 13628 / NBRC 14792 / USDA 110)
	15	224.86	Chaperone protein DnaK	Azorhizobium caulinodans (strain ATCC 43989 / DSM 5975 / JCM 20966 / NBRC 14845 /
	16	219.46	ABC transporter ATP binding protein	Bradyrhizobium diazoefficiens (strain JCM 10833 / IAM 13628 / NBRC 14792 / USDA 110)
	17	216.17	Bll6026 protein	Bradyrhizobium diazoefficiens (strain JCM 10833 / IAM 13628 / NBRC 14792 / USDA 110)
	18	213.1	Formaldehyde activating enzyme	Hyphomicrobium denitrificans (strain ATCC 51888 / DSM 1869 / NCIB 11706 / TK 0415)
	19	212.75	Phasin	Dechloromonas aromatica (strain RCB)
	20	212.45	Elongation factor Tu	Dechloromonas aromatica (strain RCB)
NH_4^+ + Urea	1	1132.34	Formaldehyde activating enzyme	Hyphomicrobium denitrificans (strain ATCC 51888 / DSM 1869 / NCIB 11706 / TK 0415)
	2	1132.34	Formaldehyde activating enzyme	Hyphomicrobium denitrificans (strain ATCC 51888 / DSM 1869 / NCIB 11706 / TK 0415)
	3	983.89	Formaldehyde activating enzyme	Hyphomicrobium denitrificans (strain ATCC 51888 / DSM 1869 / NCIB 11706 / TK 0415)
	4	422.18	PQQ dependent dehydrogenase, methanol/ethanol	Hyphomicrobium denitrificans (strain ATCC 51888 / DSM 1869 / NCIB 11706 / TK 0415)
	5	404.26	Methanol dehydrogenase large subunit like protein	Bradyrhizobium diazoefficiens (strain JCM 10833 / IAM 13628 / NBRC 14792 / USDA 110)
	6	404.26	PQQ dependent dehydrogenase, methanol/ethanol	Hyphomicrobium denitrificans (strain ATCC 51888 / DSM 1869 / NCIB 11706 / TK 0415)
	7	362.71	Flagellin	Leptothrix cholodnii (strain ATCC 51168 / LMG 8142 / SP 6)
	8	354.13	hypothetical protein AY	Gaiella sp. SCGC AG 212 M14
	9	340.41	Methanol dehydrogenase large subunit like protein	Bradyrhizobium diazoefficiens (strain JCM 10833 / IAM 13628 / NBRC 14792 / USDA 110)
	10	336.89	PQQ dependent dehydrogenase, methanol/ethanol	Hyphomicrobium denitrificans (strain ATCC 51888 / DSM 1869 / NCIB 11706 / TK 0415)
	11	334.67	Methanol dehydrogenase large subunit like protein	Bradyrhizobium diazoefficiens (strain JCM 10833 / IAM 13628 / NBRC 14792 / USDA 110)
	12	328.65	Ethanolamine utilization protein EutM	Escherichia coli (strain K12)
	13	323.2	Methanol dehydrogenase large subunit like protein	Bradyrhizobium diazoefficiens (strain JCM 10833 / IAM 13628 / NBRC 14792 / USDA 110)
	14	309.62	ATP synthase subunit beta	Dyadobacter fermentans (strain ATCC 700827 / DSM 18053 / NS114)
	15	280.84	Formaldehyde activating enzyme	Hyphomicrobium denitrificans (strain ATCC 51888 / DSM 1869 / NCIB 11706 / TK 0415)
	16	252.44	Formaldehyde activating enzyme	Hyphomicrobium denitrificans (strain ATCC 51888 / DSM 1869 / NCIB 11706 / TK 0415)
	17	203.95	ATP synthase subunit beta	Dyadobacter fermentans (strain ATCC 700827 / DSM 18053 / NS114)
	18	194.65	ATP synthase subunit beta	Dyadobacter fermentans (strain ATCC 700827 / DSM 18053 / NS114)
	19	194.2	Methanol dehydrogenase large subunit like protein	Bradyrhizobium diazoefficiens (strain JCM 10833 / IAM 13628 / NBRC 14792 / USDA 110)
	20	188.69	DNA directed RNA polymerase subunit beta	Alkalilimnicola ehrlichii (strain ATCC BAA 1101 / DSM 17681 / MLHE 1)
